# Supplementary figures and images for: Identification of key genes involved in secondary metabolite biosynthesis in Digitalis purpurea
Source: PLoS One. 2023 Mar 9;18(3):e0277293. doi: 10.1371/journal.pone.0277293 (PMC9997893; doi:10.1371/journal.pone.0277293)

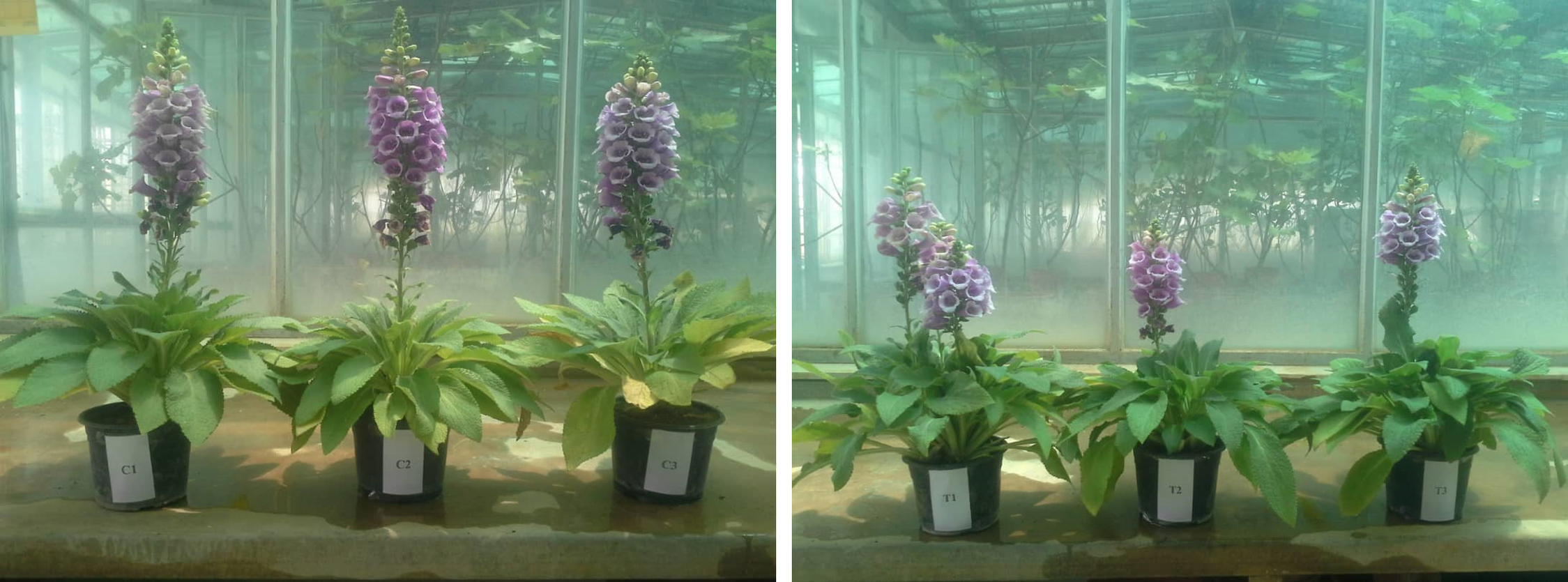

Supplement: S1 Fig — The plants were treated with 100 μM MeJA (plus 0.1% Tween-20) in 0.1% ethanol. In addition, the controls were sprayed and watered with 0.1% Tween-20 in 0.1% ethanol. The leaf samples were collected at 3, 6, 24, and 48 hours after treatment. (TIF) [file pone.0277293.s001.tif]

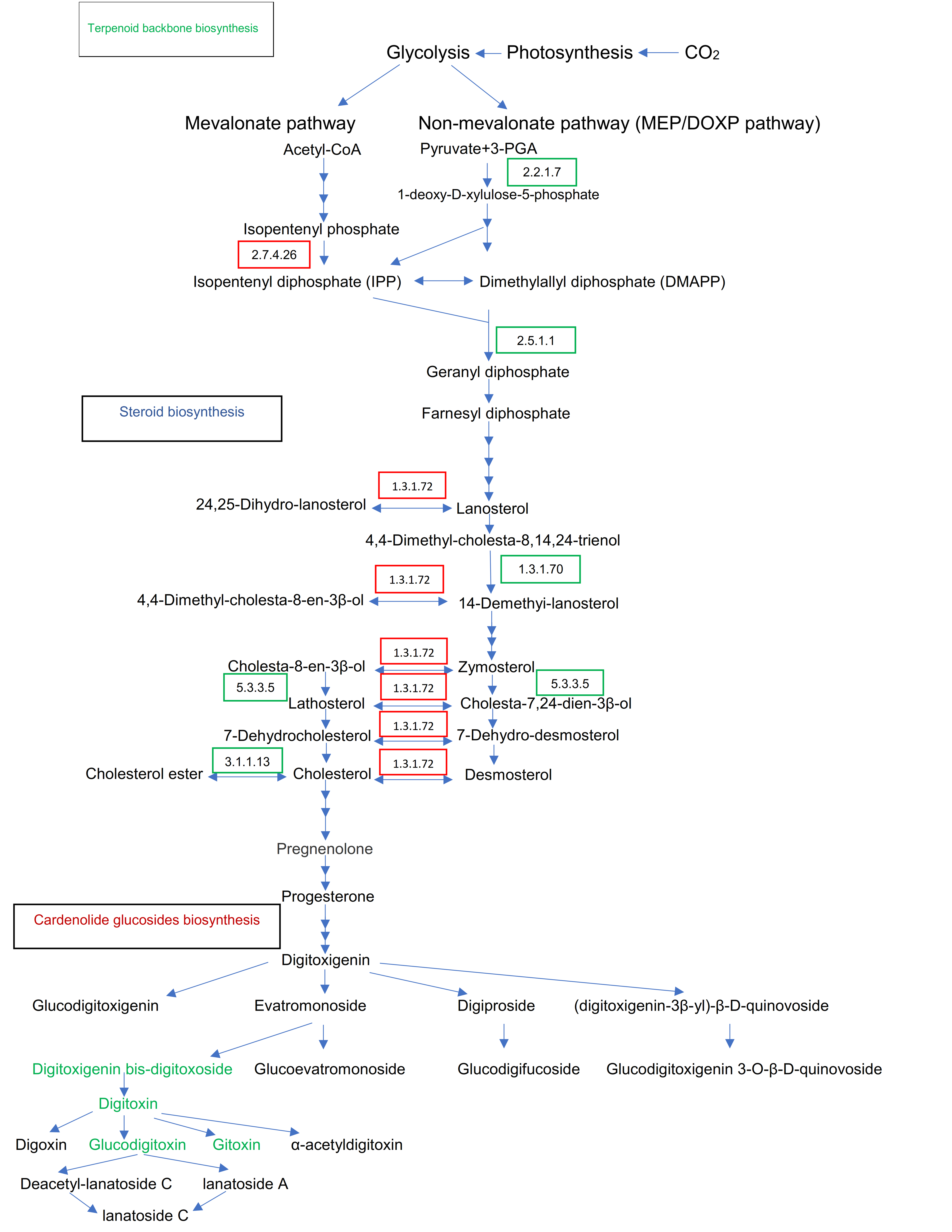

Supplement: S2 Fig — IPK with EC number 2.7.4.26 is shown in red box; is a hub gene and DWF1 with EC number 1.3.1.72 is shown in red box, is a hub gene with a great role in compound conversion. Some genes shown in green box including DXS1 with EC number 2.2.1.7, GPS1 with EC number 2.5.1.1, FK with EC number 1.3.1.70, HYD1 with EC number 5.3.3.5, and, SDP1 with EC number 3.1.1.13 that were identified in selected modules showed key roles in biosynthesis of main secondary metabolites. All of these genes are involved in the biosynthetic pathway leading to the production of cardiac glycosides. (TIF) [file pone.0277293.s002.tif]
