# Supplementary material for: Identification of key genes involved in secondary metabolite biosynthesis in Digitalis purpurea
Source: PLoS One. 2023 Mar 9;18(3):e0277293. doi: 10.1371/journal.pone.0277293 (PMC9997893; doi:10.1371/journal.pone.0277293)
Supplement: S1 Table — The metabolome datasets measured by Liquid Chromatography/Time-Of-Flight/Mass Spectrometry (LC/TOF/MS) method that were retrieved from Plant/Eukaryotic and Microbial Systems Resource database (MPR, http://metnetweb.gdcb.iastate.edu/PMR/). (DOCX) [file pone.0277293.s003.docx]

**S1 Table. The metabolome datasets of *D. purpurea.*** The metabolome datasets measured by Liquid Chromatography/Time-Of-Flight/Mass Spectrometry (LC/TOF/MS) method that were retrieved from Plant/Eukaryotic and Microbial Systems Resource database (PMR, http://metnetweb.gdcb.iastate.edu/PMR/).

| **Metabolite/Tissue** | **Digitoxigenin bis-digitoxoside** | **Glucodigitoxin** | **Gitoxin** | **Digitoxin** | **Strospeside** |
| --- | --- | --- | --- | --- | --- |
| Mature Flower | 1683.96 | 51.86 | 0.49 | 23.29 | 57.93 |
| Immature Flower | 2579.147 | 103.63 | 1.3 | 51.71333 | 1.3 |
| Sepals Mature Flower | 3522.275 | 2 | 2 | 181.8083 | 2 |
| Sepals Immature Flower | 3315.108 | 3.375 | 3.375 | 3.375 | 3.375 |
| Immature Leaf | 403.042 | 690.8711 | 0.3 | 71.97111 | 258.52 |
| Young Leaf | 1058.054 | 2005.661 | 0.385714 | 371.1143 | 203.3032 |
| Young Leaf Petiole | 2272.133 | 1131.62 | 0.642857 | 0.642857 | 0.642857 |
| Mature Leaf Petiole | 1824.199 | 576.3273 | 0.568452 | 0.568452 | 106.378 |
