# Supplementary material for: Identification of key genes involved in secondary metabolite biosynthesis in Digitalis purpurea
Source: PLoS One. 2023 Mar 9;18(3):e0277293. doi: 10.1371/journal.pone.0277293 (PMC9997893; doi:10.1371/journal.pone.0277293)
Supplement: S2 Table — (DOCX) [file pone.0277293.s004.docx]

**S2 Table. The correlation and *P-value* of candidate modules associated with the secondary metabolites.**

| **Module** | **Secondary metabolites** | **Positive correlation** | ***P-value*** |
| --- | --- | --- | --- |
| coral3 | Strospeside | 0.63 | 0.09 |
| lightsteelblue | Digitoxigenin bis-digitoxoside | 0.64 | 0.09 |
| darkorange2 | Digitoxigenin bis-digitoxoside | 0.68 | 0.07 |
| coral4 | Gitoxin | 0.69 | 0.06 |
| blue2 | Gitoxin | 0.72 | 0.04 |
| lightpink4 | Glucodigitoxin | 0.73 | 0.04 |
| blue2 | Digitoxigenin bis-digitoxoside | 0.74 | 0.04 |
| coral3 | Glucodigitoxin | 0.77 | 0.03 |
| coral4 | Digitoxigenin bis-digitoxoside | 0.79 | 0.02 |
| chocolate3 | Digitoxigenin bis-digitoxoside | 0.84 | 0.01 |
