# Supplementary material for: Identification of key genes involved in secondary metabolite biosynthesis in Digitalis purpurea
Source: PLoS One. 2023 Mar 9;18(3):e0277293. doi: 10.1371/journal.pone.0277293 (PMC9997893; doi:10.1371/journal.pone.0277293)
Supplement: S3 Table — (DOCX) [file pone.0277293.s005.docx]

**S3 Table. Some genes in selected modules with central roles in biosynthesis of secondary metabolites.**

| **Modules** | **Sequence ID** | **Accession** | **Pathways** |
| --- | --- | --- | --- |
| coral3 | G33710i1L757 | AT4G15560.1 | ath00900: Terpenoid backbone biosynthesis |
|  | G11783i4L1468 | AT2G34630.2 | ath00900: Terpenoid backbone biosynthesis |
|  | G483i4L1413 | AT3G52940.1 | ko00100: Steroid biosynthesis |
|  | G48583i1L407 | AT3G19820.3 | ko00100: Steroid biosynthesis |
|  | G4455i1L1908 | AT3G10230.1 | ath00906: Carotenoid biosynthesis |
|  | G24312i1L1027 | AT4G02780.1 | ath00904: Diterpenoid biosynthesis |
|  | G19585iso1L383 | AT5G07990.1 | ath00941: Flavonoid biosynthesis |
|  | G19585iso1L383 | AT5G07990.1 | ath00944: Flavone and flavanol biosynthesis |
|  | G15282i1L1692 | AT5G23960.2 | ath00909: Sesquiterpenoid and triterpenoid biosynthesis |
| darkorange2 | G5726i4L1080 | AT1G20050.1 | ath00100: Steroid biosynthesis |
| lightpink4 | G6584i2L1435 | AT5G50375.2 | ath00100: Steroid biosynthesis |
| lightsteelblue | G53803i1L1366 | AT5G04040.2 | ath00100: Steroid biosynthesis |
| chocolate3 | G4422i1L1614 | AT1G26640.1 | ath00900: Terpenoid backbone biosynthesis |
