# Supplementary material for: Identification of key genes involved in secondary metabolite biosynthesis in Digitalis purpurea
Source: PLoS One. 2023 Mar 9;18(3):e0277293. doi: 10.1371/journal.pone.0277293 (PMC9997893; doi:10.1371/journal.pone.0277293)
Supplement: S4 Table — (DOCX) [file pone.0277293.s006.docx]

**S4 Table. Transcription factors involved in the modules related to the production of secondary metabolites.**

| **Module** | **Sequence ID** | **Transcription Factors** |
| --- | --- | --- |
| blue2 | G10637i2L1100-2R | B3 |
|  | G501i9L2189-0F | bHLH |
|  | G10419i1L395-0R | C2H2 |
|  | G9709i2L1064-0R | C2H2 |
|  | G3254i9L2221-0F | GRAS |
|  | G6433i4L1373-0F | MYB |
|  | G16631i2L1735-1R | Trihelix |
| chocolate3 | G5195i3L1021-0F | B3 |
|  | G3077i1L870-0F | C2H2 |
|  | G38924i1L1068-1F | MYB |
|  | G12216i4L1914-1F | Trihelix |
| darkorange2 | G42957i1L750-0F | AP2/ERF-ERF |
|  | G6434i3L1972-2R | C2H2 |
|  | G17981i1L401-0F | FAR1 |
|  | G34370i4L1624-0F | GARP-G2-like |
|  | G7709i1L846-1F | HSF |
|  | G18584i1L1216-1R | NAC |
|  | G8453i2L1777-2F | SBP |
| coral3 | G16222i3L3467-0R | AP2/ERF-ERF |
|  | G2809i8L2658-2F | AP2/ERF-ERF |
|  | G5516i3L1106-0F | B3 |
|  | G9180i5L2214-2F | B3-ARF |
|  | G14815i9L2416-1R | bHLH |
|  | G6167i3L2423-2R | bHLH |
|  | G7863i1L1743-1R | bHLH |
|  | G14386i7L834-2F | bZIP |
|  | G1457i2L1693-2R | bZIP |
|  | G17209i5L862-1F | bZIP |
|  | G2703i4L1609-0F | bZIP |
|  | G3089i9L1382-0R | bZIP |
|  | G7952i3L1405-0R | C2C2-Dof |
|  | G3834i9L2236-2F | C2C2- GATA |
|  | G5503i1L2336-1F | C2C2- GATA |
|  | G93155i1L538-0R | C2C2- GATA |
|  | G4821i4L1414-0F | C2H2 |
|  | G99845i1L290-2R | GARP-G2-like |
|  | G18633i1L951-1F | GRAS |
|  | G2966i5L2038-1R | GRAS |
|  | G68417i1L660-0R | GRAS |
|  | G6955i1L1407-1F | GRAS |
|  | G3911i8L1455-1R | HB-KNOX |
|  | G21081i1L729-0R | LOB |
|  | G26266i1L638-2F | LOB |
|  | G26855i1L558-0F | LOB |
|  | G39044i1L605-0F | MADS-M-type |
|  | G4465i2L2160-2F | MADS-MIKC |
|  | G5721i3L1232-0F | MADS-MIKC |
|  | G10166i2L1725-2F | MYB |
|  | G12558i2L909-0F | MYB |
|  | G15723i2L1122-0F | MYB |
|  | G6033i3L1293-0R | MYB |
|  | G695i6L1437-0F | MYB |
|  | G14680i3L1613-0R | MYB-related |
|  | G14680i3L1613-2R | MYB-related |
|  | G23976i1L615-1F | MYB-related |
|  | G6742i7L1167-0F | NF-YB |
|  | G18424i8L2444-2F | TCP |
|  | G5813i4L1677-2F | Tify |
|  | G6910i4L1243-1R | Tify |
|  | G10890i2L1189-1R | Trihelix |
|  | G11819i3L2031-0F | WRKY |
|  | G6833i9L1495-1F | WRKY |
|  | G7498i5L2242-2R | WRKY |
| lightpink4 | G21099i2L776-1R | AP2/ERF-ERF |
|  | G24594i1L739-0F | AP2/ERF-ERF |
|  | G47816i1L989-2R | AP2/ERF-ERF |
|  | G15639i2L1041-2F | bHLH |
|  | G15796i1L723-1R | bHLH |
|  | G80974i1L672-2F | bHLH |
|  | G103943i1L292-2F | C2C2-Dof |
|  | G14168i7L1141-2F | C2C2-Dof |
|  | G1275i1L1397-0R | C2C2-GATA |
|  | G14284i1L1770-1F | C2C2-GATA |
|  | G7652i5L1210-1F | C2H2 |
|  | G26238i7L1666-1F | GARP-G2-like |
|  | G26513i1L657-0F | HB-HD-ZIP |
|  | G26378i1L512-0F | HB-other |
|  | G6607i9L1123-1F | WRKY |
| lightsteelblue | G18658i1L991-1R | bHLH |
|  | G17896i3L1968-1R | bZIP |
|  | G15017i4L1209-2F | bZIP |
|  | G7213i1L1759-1R | C3H |
|  | G40514i1L525-1F | EIL |
|  | G8090i9L1728-1F | GRF |
|  | G6013i6L1486-1R | MADS-MIKC |
|  | G8443i3L1564-1F | MYB |
|  | G100456i1L403-2R | TCP |
|  | G5163i8L1535-0F | Tify |
|  | G15539i2L1727-2F | WRKY |
