# Supplementary material for: Identification of key genes involved in secondary metabolite biosynthesis in Digitalis purpurea
Source: PLoS One. 2023 Mar 9;18(3):e0277293. doi: 10.1371/journal.pone.0277293 (PMC9997893; doi:10.1371/journal.pone.0277293)
Supplement: S5 Table — (DOCX) [file pone.0277293.s007.docx]

**S5 Table. Transcriptional regulators in the modules related to secondary metabolite production.**

| **Module** | **Sequence ID** | **Transcriptional Regulators** |
| --- | --- | --- |
| blue2 | G13327i1L978-0F | GNAT |
|  | G263i6L2362-1R | GNAT |
| chocolate3 | G1135i10L1195-1F | AUX/IAA |
|  | G28524i1L741-0R | AUX/IAA |
| coral3 | G2372i7L1517-0F | AUX/IAA |
|  | G4056i5L1409-1R | GNAT |
|  | G3433i4L1553-2F | IWS1 |
|  | G12298i6L1860-0R | mTERF |
|  | G12298i6L1860-2R | mTERF |
|  | G4420i1L1966-0F | mTERF |
|  | G5589i1L1640-0R | mTERF |
|  | G6142i6L3089-1R | mTERF |
|  | G5946i1L2185-1R | PHD |
|  | G136i5L2477-0F | Pseudo ARR-B |
|  | G1161i3L1719-0R | SET |
|  | G5777i6L1906-0F | SWI/SNF-BAF60b |
|  | G5359i8L2787-0F | TRAF |
| darkorange2 | G5376i6L1603-2R | AUX/IAA |
|  | G5098i6L1129-0R | Others |
|  | G17849i1L633-2R | SWI/SNF-SWI3 |
| lightpink4 | G32526i1L785-2R | AUX/IAA |
|  | G37885i2L735-1F | GNAT |
