# Supplementary material for: Identification of key genes involved in secondary metabolite biosynthesis in Digitalis purpurea
Source: PLoS One. 2023 Mar 9;18(3):e0277293. doi: 10.1371/journal.pone.0277293 (PMC9997893; doi:10.1371/journal.pone.0277293)
Supplement: S6 Table — (DOCX) [file pone.0277293.s008.docx]

**S6 Table. Protein kinases involved to the biosynthesis of secondary metabolites.**

| **Module** | **Sequence ID** | **Protein Kinases** |
| --- | --- | --- |
| blue2 | G24307i2L975-0R | RLK-Pelle_LRR-II |
|  | G434i1L1247-2F | RLK-Pelle_LysM |
| chocolate3 | G1166i4L2020-0F | RLK-Pelle_L-LEC |
| coral3 | G81692i1L279-0F | CAMK_CDPK |
|  | G16514i1L1564-0R | CAMK_CAMKL-CHK1 |
|  | G16514i1L1564-2R | CAMK_CAMKL-CHK1 |
|  | G2111i9L2264-1F | CMGC_SRPK |
|  | G2174i8L1782-0R | CMGC_MAPK |
|  | G1511i10L1881-1F | CMGC_GSK |
|  | G11249i8L2474-1F | RLK-Pelle_LRR-Xa |
|  | G9923i1L730-2F | RLK-Pelle_LRR-XI-1 |
|  | G12801i1L2106-0R | RLK-Pelle_DLSV |
|  | G13961i9L2697-2F | RLK-Pelle_DLSV |
|  | G6042i7L3280-1F | RLK-Pelle_DLSV |
|  | G7068i1L1461-0F | RLK-Pelle_DLSV |
|  | G13725i1L1742-2F | RLK-Pelle_LRK10L-2 |
|  | G8753i3L2664-1R | RLK-Pelle_RLCK-XII-1 |
|  | G8917i2L2053-2R | RLK-Pelle_RLCK-VI |
|  | G2529i2L1671-0R | RLK-Pelle_RLCK-XV |
|  | G3460i7L2322-1F | RLK-Pelle_RLCK-V |
|  | G35242i1L346-0F | RLK-Pelle_WAK |
|  | G7808i2L2542-2F | RLK-Pelle_WAK_LRK10L-1 |
|  | G9269i1L1533-1F | RLK-Pelle_WAK |
|  | G10240i3L2343-0R | STE_STE11 |
|  | G14412i2L912-1F | STE_STE20-Fray |
|  | G4836i2L1586-0F | TKL-Pl-4 |
| coral4 | G15784i1L1657-2F | RLK-Pelle_WAK |
|  | G9176i1L1443-0F | RLK-Pelle_RLCK-VIIa-2 |
| darkorange2 | G10716i7L2534-2F | CMGC_CK2 |
|  | G12318i2L2419-0R | RLK-Pelle_L-LEC |
|  | G18755i2L885-1F | STE_STE11 |
| lightpink4 | G11359i1L2310-1F | RLK-Pelle_LRR-IX |
|  | G9678i1L1281-2R | RLK-Pelle_RLCK-XIII |
| lightsteelblue | G1152i9L2038-0R | CAMK_CAMKL-CHK1 |
|  | G15729i1L1450-0R | RLK-Pelle_RLCK-X |
|  | G15729i1L1450-2R | RLK-Pelle_RLCK-X |
|  | G15084i4L1968-1F | RLK-Pelle_RLCK-VIIa-2 |
