# Supplementary material for: Identification of key genes involved in secondary metabolite biosynthesis in Digitalis purpurea
Source: PLoS One. 2023 Mar 9;18(3):e0277293. doi: 10.1371/journal.pone.0277293 (PMC9997893; doi:10.1371/journal.pone.0277293)
Supplement: S7 Table — List of protein transporters in modules related to secondary metabolite production. (DOCX) [file pone.0277293.s009.docx]

**S7 Table.** **Protein transporters.** List of protein transporters in modules related to secondary metabolite production.

| **Module** | **Family of transporters** | **Number** |
| --- | --- | --- |
| blue2 | The Chloroplast Envelope Protein Translocase (CEPT or Tic-Toc) Family | 1 |
|  | The Amino Acid/Auxin Permease (AAAP) Family | 1 |
|  | The Domain of Unknown Function 3339(DUF3339) Family | 1 |
|  | The ATP-binding Cassette (ABC) Superfamily | 2 |
|  | The Divalent Anion: Na^+^ Symporter (DASS) Family | 2 |
|  | The Major Facilitator Superfamily (MFS) | 1 |
|  | The Bile Acid: Na^+^ Symporter (BASS) Family | 1 |
|  | The Mechanosensitive Calcium Channel (MCA) Family | 1 |
|  | The Octameric Exocyst (Exocyst) Family | 1 |
| chocolate3 | The H^+^ or Na^+^-translocating NADH Dehydrogenase (NDH) Family | 1 |
|  | The Secretory Carrier-Associated Membrane Protein (SCAMP) Family | 1 |
|  | The Mechanosensitive Calcium Channel (MCA) Family | 3 |
|  | The Major Intrinsic Protein (MIP) Family | 1 |
|  | The Peroxisomal Protein Importer (PPI) Family | 1 |
|  | The Proton-translocating Quinol:Cytochrome c Reductase (QCR) Superfamily | 1 |
|  | The Nucleobase/Ascorbate Transporter (NAT) or Nucleobase:Cation Symporter-2 (NCS2) Family | 1 |
|  | The Eukaryotic Nuclear Pore Complex (E-NPC) Family | 1 |
|  | The Major Facilitator Superfamily (MFS) | 1 |
|  | The Domain of Unknown Function 3339 (DUF3339) Family | 1 |
| coral3 | The Autophagy-related Phagophoreformation Transporter (APT) Family | 3 |
|  | The Mitochondrial Carrier (MC) Family | 5 |
|  | The Domain of Unknown Function 3339 (DUF3339) Family | 2 |
|  | The Plant Photosystem I Supercomplex (PSI) Family | 1 |
|  | The Presenilin ER Ca^2+^Leak Channel (Presenilin)Family | 1 |
|  | The Proton-translocating Quinol:Cytochrome c Reductase (QCR) Superfamily | 1 |
|  | The Amino Acid/Auxin Permease (AAAP) Superfamily | 1 |
|  | The H^+^- or Na^+^-translocating F-type, V-type and A-type ATPase(F-ATPase) Superfamily | 5 |
|  | The Cation Diffusion Facilitator (CDF) Family | 2 |
|  | The Eukaryotic Nuclear Pore Complex (E-NPC) Family | 4 |
|  | The Retromer Assembly Apparatus (Retromer AA) Family | 3 |
|  | The G-protein-coupled receptor (GPCR) Family | 1 |
|  | The Drug/Metabolite Transporter (DMT) Superfamily | 6 |
|  | The Endoplasmic Reticulum Surface Retrieval Pathway (ER-SURF) for Mitochondrial Membrane Protein Targeting | 1 |
|  | The Klotho Auxiliary Protein (Klotho) Family | 1 |
|  | The Mechanosensitive Calcium Channel (MCA) Family | 15 |
|  | The Retromer-dependent Vacuolar Protein Sorting (R-VPS) Family | 1 |
|  | The Plasmodium Translocon of Exported proteins (PTEX) Family | 1 |
|  | The Polyoma Virus SV40 ER Penitration Channel (VPEC) Family | 1 |
|  | The Basigin (Basigin) Family | 1 |
|  | The Curature-stabilizing Protein YOP Family | 1 |
|  | The ATP-binding Cassette (YOP1) Family | 6 |
|  | The Endosomal Sorting Complexes Required for Transport III(ESCRT-III) Family | 1 |
|  | The Membrane Protein Insertase (YidC/Alb3/Oxa1) Family | 1 |
|  | The Phosphate Permease (Pho1) Family | 1 |
|  | The Mitochondrial and Plastid Porin (MPP) Family | 1 |
|  | The Bactericidal Permeability-Increasing Protein (BPIP) Family | 1 |
|  | The Reticulon (Reticulon) Family | 1 |
|  | The Membrane Mg^2+^ Transporter (MMgT) Family | 2 |
|  | The Proton-dependent Oligopeptide Transporter (POT/PTR) Family | 2 |
|  | The Peroxisomal Protein Importer (PPI) Family | 1 |
|  | The Eukaryotic Cytochrome b561 (Cytb561) Family | 1 |
|  | The P-type ATPase(P-ATPase) Superfamily | 2 |
|  | The Cholesterol Uptake Protein (ChUP) or Double Stranded RNA Uptake Family | 1 |
|  | The CorA Metal Ion Transporter (MIT) Family | 1 |
|  | The Metal Ion (Mn^2+^-iron) Transporter (Nramp) Family | 1 |
|  | The Multidrug/Oligosaccharidyl-lipid/Polysaccharide (MOP) Flippase Superfamily | 4 |
|  | The Flavin-based Extracellular Electron Transfer(F-EET) Family | 1 |
|  | The Sulfate Permease (SulP) Family | 1 |
|  | The Folate-Biopterin Transporter (FBT) Family | 1 |
|  | The Calmodulin Calcium Binding Protein (Calmodulin) Family | 1 |
|  | The Sweet; PQ-loop; Saliva; MtN3 (Sweet) Family | 2 |
|  | The Oligopeptide Transporter (OPT) Family | 1 |
|  | The Annexin (Annexin) Family | 1 |
|  | The Major Intrinsic Protein (MIP) Family | 1 |
|  | The Glutamate-gated Ion Channel (GIC) Family of Neurotransmitter Receptors | 1 |
|  | The Ammonium Channel Transporter (Amt) Family | 1 |
|  | The Transferrin Receptor, CD71, (TFR) Family | 1 |
|  | The Mitochondrial ATP synthase Stress-responsive Protein (MASP) Family | 2 |
|  | The Gap Junction-forming Innexin (Innexin) Family | 1 |
|  | The Pock Size-determining Protein (PSDP) Family | 1 |
|  | The K+ Uptake Permease (KUP) Family | 1 |
|  | The Chloroplast Envelope Protein Translocase (CEPT or Tic-Toc) Family | 1 |
|  | The Epithelial Chloride Channel (E-ClC) Family | 1 |
|  | The Zinc (Zn^2+^)-Iron (Fe^2+^) Permease (ZIP) Family | 1 |
| coral4 | The Mg^2+^ /Ca^2+^ Uniporter (MCU) Family | 1 |
|  | The Drug/Metabolite Transporter (DMT) Superfamily | 1 |
|  | The Mechanosensitive Calcium Channel (MCA) Family | 2 |
|  | The Fatty Acid Group Translocation (FAT) Family | 1 |
|  | The Endoplasmic Reticular Retrotranslocon (ER-RT or ERAD) Family | 1 |
|  | The ATP-binding Cassette Superfamily | 1 |
|  | The Proton-translocating Cytochrome Oxidase (COX) Superfamily | 1 |
| darkorange2 | The Huntington-interacting protein 14 (HIP14) Family | 1 |
|  | The Vitamin A Receptor/Transporter (STRA6) Family | 1 |
|  | The 14-3-3 protein (14-3-3) Family | 1 |
|  | The Eukaryotic Nuclear Pore Complex (E-NPC) Family | 2 |
|  | The Major Facilitator Superfamily (MFS) | 2 |
|  | The Drug/Metabolite Transporter (DMT) Superfamily | 1 |
|  | The Retromer-dependent Vacuolar Protein Sorting (R-VPS) Family | 1 |
|  | The Mechanosensitive Calcium Channel (MCA) Family | 2 |
|  | The Eukaryotic Cytochrome b561 (Cytb561) Family | 1 |
|  | The Cation Channel-forming Heat Shock Protein-70 (Hsp70) Family | 1 |
|  | The Ferroportin (Fpn) Family | 1 |
|  | The Domain of Unknown Function 3339 (DUF3339) Family | 1 |
|  | The ATP-binding Cassette (ABC) Superfamily | 2 |
|  | The Endosomal Sorting Complexes Required for Transport III (ESCRT-III) Family | 1 |
|  | The Multidrug/Oligosaccharidyl-lipid/Polysaccharide (MOP) Flippase Superfamily | 1 |
|  | The Extended-Synaptotagmin (E-Syt) Family | 1 |
| lightpink4 | The Membrane Contact Site (MCS) Family | 1 |
|  | The Sweet; PQ-loop; Saliva; MtN3 (Sweet) Family | 1 |
|  | The Endoplasmic Reticular Retrotranslocon (ER-RT or ERAD) Family | 4 |
|  | The Multidrug/Oligosaccharidyl-lipid/Polysaccharide (MOP) Flippase Superfamily | 2 |
|  | The AAA-ATPase, Bcs1 (Bcs1) Family | 1 |
|  | The Domain of Unknown Function 3339 (DUF3339) Family | 1 |
|  | The Plant Plasmodesmata (PPD) Family | 2 |
|  | The Putative Tripartite Zn2 Transporter (TZT) Family | 1 |
|  | The H^+^- or Na^+^-translocating F-type, V-type and A-type ATPase (F-ATPase) Superfamily | 1 |
|  | The P-type ATPase (P-ATPase) Superfamily | 1 |
|  | The Mechanosensitive Calcium Channel (MCA) Family | 2 |
|  | The Na^+^-transporting Carboxylic Acid Decarboxylase (NaT-DC) Family | 1 |
|  | The Auxin Efflux Carrier (AEC) Family | 1 |
|  | The Eukaryotic Nuclear Pore Complex (E-NPC) Family | 1 |
|  | The Tellurite-resistance/Dicarboxylate Transporter (TDT) Family | 1 |
| lightsteelblue | The Drug/Metabolite Transporter (DMT) Superfamily | 1 |
|  | The Sulfate Permease (SulP) Family | 2 |
|  | The Peroxisomal Protein Importer (PPI) Family | 1 |
|  | The Pock Size-determining Protein (PSDP) Family | 1 |
|  | The Lysosomal Cystine Transporter (LCT) Family | 1 |
|  | The Sweet; PQ-loop; Saliva; MtN3 (Sweet) Family | 1 |
|  | The Ezrin/Radixin/Moesin-binding Phosphoprotein 50 (EBP50) Family | 1 |
|  | The ATP-binding Cassette (ABC) Superfamily | 1 |
|  | The gp91phox Phagocyte NADPH Oxidase-associated Cytochrome b558 (Phox) Family | 2 |
|  | The Eukaryotic Cytochrome b561 (Cytb561) Family | 1 |
|  | The Endoplasmic Reticulum Surface Retrieval Pathway (ER-SURF) for Mitochondrial Membrane Protein Targeting | 1 |
|  | The Major Intrinsic Protein (MIP) Family | 1 |
|  | The Amino Acid/Auxin Permease (AAAP) Family | 1 |
|  | The Major Facilitator Superfamily (MFS) | 2 |
|  | The Mechanosensitive Calcium Channel (MCA) Family | 2 |
|  | The Cation Diffusion Facilitator (CDF) Family | 1 |
|  | The Endoplasmic Reticulum Retention Receptor (KDELR) Family | 1 |
|  | The Calmodulin Calcium Binding Protein (Calmodulin) Family | 1 |
