# Supplementary material for: Identification of key genes involved in secondary metabolite biosynthesis in Digitalis purpurea
Source: PLoS One. 2023 Mar 9;18(3):e0277293. doi: 10.1371/journal.pone.0277293 (PMC9997893; doi:10.1371/journal.pone.0277293)
Supplement: S8 Table — (DOCX) [file pone.0277293.s010.docx]

**S8 Table. The key mlncRNAs identified in the coral3 module.**

| **Sequence ID** | **Accession** | **Description** | **Authors** |
| --- | --- | --- | --- |
| G44107i1L307 | JO461863 | TSA: *Digitalis purpurea* contig02067.Dipuleaves mRNA sequence | (Wu *et al.,* 2012) |
| G86604i1L338 | JO460006 | TSA: *Digitalis purpurea* contig00004.Dipuleaves mRNA sequence | (Wu *et al.,* 2012) |
| G88908i1L331 | JO466327 | TSA: *Digitalis purpurea* contig06812.Dipuleaves mRNA sequence | (Wu *et al.,* 2012) |
| G104840i1L393 | JO462746 | TSA: *Digitalis purpurea* contig03010.Dipuleaves mRNA sequence | (Wu *et al.,* 2012) |
