# Supplementary material for: Identification of key genes involved in secondary metabolite biosynthesis in Digitalis purpurea
Source: PLoS One. 2023 Mar 9;18(3):e0277293. doi: 10.1371/journal.pone.0277293 (PMC9997893; doi:10.1371/journal.pone.0277293)
Supplement: S9 Table — The top 20 hub genes in the networks of each selected module, ranked by the MCC method. (DOCX) [file pone.0277293.s011.docx]

**S9 Table. The top 140 hub genes.** The top 20 hub genes in the networks of each selected module, ranked by the MCC method.

| **Module** | **Name** | **Score** | **Rank** |
| --- | --- | --- | --- |
| blue2 | G507i4L1514 | 90 | 1 |
|  | G3254i9L2221 | 81 | 2 |
|  | G5051i5L1826 | 80 | 3 |
|  | G16631i2L1735 | 78 | 4 |
|  | G13596i1L492 | 76 | 5 |
|  | G3462i1L1390 | 42 | 6 |
|  | G6516i3L2956 | 30 | 7 |
|  | G13670i3L1010 | 30 | 7 |
|  | G8924i8L1475 | 30 | 7 |
|  | G12261i1L1230 | 24 | 10 |
|  | G15432i1L463 | 24 | 10 |
|  | G16598i1L927 | 15 | 12 |
|  | G100888i1L301 | 14 | 13 |
|  | G35880i1L684 | 13 | 14 |
|  | G43154i1L567 | 12 | 15 |
|  | G54986i1L601 | 10 | 16 |
|  | G101344i1L379 | 9 | 17 |
|  | G4169i1L1419 | 9 | 17 |
|  | G42111i1L1561 | 8 | 19 |
|  | G64486i1L292 | 8 | 19 |
| chocolate3 | G19329i1L356 | 264 | 1 |
|  | G5309i6L1104 | 258 | 2 |
|  | G3077i1L870 | 249 | 3 |
|  | G25428i2L733 | 248 | 4 |
|  | G46369i2L448 | 248 | 4 |
|  | G20448i2L620 | 129 | 6 |
|  | G4422i1L1614 | 121 | 7 |
|  | G19633i1L273 | 26 | 8 |
|  | G81888i1L295 | 14 | 9 |
|  | G94067i1L1035 | 13 | 10 |
|  | G12980i1L1957 | 12 | 11 |
|  | G9927i2L384 | 12 | 11 |
|  | G740i7L1692 | 11 | 13 |
|  | G45399i1L296 | 9 | 14 |
|  | G52097i2L434 | 8 | 15 |
|  | G96728i1L338 | 8 | 15 |
|  | G19359i1L611 | 6 | 17 |
|  | G31624i3L780 | 6 | 17 |
|  | G6638i1L284 | 5 | 19 |
|  | G7238i1L1135 | 5 | 19 |
| coral3 | G100958i1L352 | 2.05E+90 | 1 |
|  | G102416i1L350 | 2.05E+90 | 1 |
|  | G102518i1L348 | 2.05E+90 | 1 |
|  | G103219i1L278 | 2.05E+90 | 1 |
|  | G18126i3L790 | 2.05E+90 | 1 |
|  | G22654i2L548 | 2.05E+90 | 1 |
|  | G24341i1L393 | 2.05E+90 | 1 |
|  | G28098i1L361 | 2.05E+90 | 1 |
|  | G33602i1L333 | 2.05E+90 | 1 |
|  | G39717i1L324 | 2.05E+90 | 1 |
|  | G50322i1L308 | 2.05E+90 | 1 |
|  | G52161i1L426 | 2.05E+90 | 1 |
|  | G65580i1L323 | 2.05E+90 | 1 |
|  | G69187i1L323 | 2.05E+90 | 1 |
|  | G73507i1L280 | 2.05E+90 | 1 |
|  | G81305i1L352 | 2.05E+90 | 1 |
|  | G84695i1L284 | 2.05E+90 | 1 |
|  | G94899i1L481 | 2.05E+90 | 1 |
|  | G95075i1L308 | 2.05E+90 | 1 |
|  | G95883i1L496 | 2.05E+90 | 1 |
| coral4 | G94673i1L362 | 1.66E+12 | 1 |
|  | G43246i1L514 | 1.66E+12 | 2 |
|  | G46465i1L522 | 1.66E+12 | 2 |
|  | G100230i1L462 | 1.66E+12 | 4 |
|  | G16183i1L537 | 1.66E+12 | 4 |
|  | G19799i1L493 | 1.66E+12 | 4 |
|  | G29117i1L421 | 1.66E+12 | 7 |
|  | G15887i1L386 | 1.66E+12 | 8 |
|  | G32707i1L356 | 1.66E+12 | 8 |
|  | G90282i1L405 | 1.66E+12 | 8 |
|  | G105887i1L345 | 1.58E+12 | 11 |
|  | G95352i1L319 | 1.58E+12 | 11 |
|  | G80897i1L281 | 1.57E+12 | 13 |
|  | G82974i1L489 | 1.48E+12 | 14 |
|  | G27740i1L452 | 1.40E+12 | 15 |
|  | G70621i1L873 | 1.39E+12 | 16 |
|  | G90369i1L310 | 2.62E+11 | 17 |
|  | G79480i1L385 | 1.75E+11 | 18 |
|  | G23205i1L481 | 9.34E+10 | 19 |
|  | G103763i1L473 | 8.72E+10 | 20 |
| darkorange2 | G16129i1L964 | 5.08E+07 | 1 |
|  | G17981i1L401 | 5.08E+07 | 1 |
|  | G29390i1L715 | 5.08E+07 | 1 |
|  | G32660i1L455 | 5.08E+07 | 1 |
|  | G36657i1L465 | 5.08E+07 | 1 |
|  | G85810i1L414 | 5.08E+07 | 1 |
|  | G101040i1L487 | 5.08E+07 | 1 |
|  | G25740i1L487 | 4.72E+07 | 8 |
|  | G12674i1L327 | 4.72E+07 | 8 |
|  | G25218i1L563 | 4.35E+07 | 10 |
|  | G18487i1L294 | 3.99E+07 | 11 |
|  | G12147i1L879 | 3.99E+07 | 11 |
|  | G38426i1L609 | 1.09E+07 | 13 |
|  | G22508i1L503 | 7257600 | 14 |
|  | G21470i1L602 | 3628800 | 15 |
|  | G26533i1L419 | 3628800 | 15 |
|  | G37632i1L289 | 762744 | 17 |
|  | G99640i1L408 | 762744 | 17 |
|  | G88596i1L396 | 762480 | 19 |
|  | G92200i1L438 | 762480 | 19 |
| lightpink4 | G17953i1L750 | 7.95E+18 | 1 |
|  | G42785i1L349 | 7.94E+18 | 2 |
|  | G26513i1L657 | 7.80E+18 | 3 |
|  | G24377i1L463 | 7.80E+18 | 4 |
|  | G37096i1L543 | 7.70E+18 | 5 |
|  | G30766i1L415 | 7.67E+18 | 6 |
|  | G38425i1L682 | 7.67E+18 | 7 |
|  | G39654i1L291 | 7.67E+18 | 8 |
|  | G13591i1L982 | 7.67E+18 | 9 |
|  | G22559i1L300 | 7.67E+18 | 9 |
|  | G28539i1L702 | 7.67E+18 | 11 |
|  | G37009i1L270 | 7.67E+18 | 12 |
|  | G39539i1L338 | 7.67E+18 | 13 |
|  | G21873i1L396 | 7.67E+18 | 14 |
|  | G23825i1L361 | 7.67E+18 | 14 |
|  | G22305i1L332 | 7.67E+18 | 16 |
|  | G64342i1L383 | 7.67E+18 | 17 |
|  | G83834i1L343 | 7.58E+18 | 18 |
|  | G42263i1L300 | 7.56E+18 | 19 |
|  | G36769i1L379 | 5.23E+18 | 20 |
| lightsteelblue | G27330i1L482 | 5.19E+07 | 1 |
|  | G39759i1L487 | 5.19E+07 | 2 |
|  | G100456i1L403 | 5.19E+07 | 3 |
|  | G101549i1L334 | 5.19E+07 | 3 |
|  | G100915i1L343 | 5.19E+07 | 5 |
|  | G20050i1L372 | 5.19E+07 | 6 |
|  | G27409i1L790 | 5.15E+07 | 7 |
|  | G103936i1L282 | 5.15E+07 | 8 |
|  | G26014i1L279 | 5.12E+07 | 9 |
|  | G66958i1L444 | 4.39E+07 | 10 |
|  | G97447i1L406 | 4.35E+07 | 11 |
|  | G98031i1L413 | 4.03E+07 | 12 |
|  | G4559i9L1760 | 8024406 | 13 |
|  | G18535i1L478 | 7257606 | 14 |
|  | G100833i1L283 | 725772 | 15 |
|  | G3578i1L1107 | 404280 | 16 |
|  | G25459i1L551 | 161304 | 17 |
|  | G27138i1L1265 | 161304 | 17 |
|  | G94161i1L316 | 161286 | 19 |
|  | G47242i2L512 | 161281 | 20 |
