# Supplementary material for: Identification of key genes involved in secondary metabolite biosynthesis in Digitalis purpurea
Source: PLoS One. 2023 Mar 9;18(3):e0277293. doi: 10.1371/journal.pone.0277293 (PMC9997893; doi:10.1371/journal.pone.0277293)
Supplement: S10 Table — (DOCX) [file pone.0277293.s012.docx]

**S10 Table. Protein-protein interactions of hub proteins.**

| **Node1** | **Sequence ID Node 1** | **Node 2** | **Sequence ID Node 2** | **Score** |
| --- | --- | --- | --- | --- |
| AT2G19730.2 | G22654i2L548 | AT3G10950.1 | G13596i1L492/ G73507i1L280 | 0.999 |
| AT2G19730.2 | G22654i2L548 | AT3G18740.1 | G25740i1L487 | 0.999 |
| AT3G10950.1 | G13596i1L492/ G73507i1L280 | AT2G19730.2 | G22654i2L548 | 0.999 |
| AT3G10950.1 | G13596i1L492/ G73507i1L280 | AT3G18740.1 | G25740i1L487 | 0.999 |
| AT3G11630.1 | G18126i3L790 | FER4 (AT2G40300.1) | G5051i5L1826 | 0.934 |
| AT3G11630.1 | G18126i3L790 | UPL2 (AT1G70320.1) | G95075i1L308 | 0.91 |
| AT3G18740.1 | G25740i1L487 | AT2G19730.2 | G22654i2L548 | 0.999 |
| AT3G18740.1 | G25740i1L487 | AT3G10950.1 | G13596i1L492/ G73507i1L280 | 0.999 |
| AT4G28060.1 | G19329i1L356 | COX2 (ATMG00160.1) | G105887i1L345 | 0.974 |
| COX2 (ATMG00160.1) | G105887i1L345 | AT4G28060.1 | G19329i1L356 | 0.974 |
| FER4 (AT2G40300.1) | G5051i5L1826 | AT3G11630.1 | G18126i3L790 | 0.934 |
| FER4 (AT2G40300.1) | G5051i5L1826 | UPL2 (AT1G70320.1) | G95075i1L308 | 0.9 |
| RHC1A (AT2G40830.2) | G25218i1L563 | UBC5 (AT1G63800.1) | G5309i6L1104 | 0.901 |
| RHC1A (AT2G40830.2) | G25218i1L563 | UBC7 (AT5G59300.1) | G70621i1L873 | 0.912 |
| RHC1A (AT2G40830.2) | G25218i1L563 | UPL2 (AT1G70320.1) | G95075i1L308 | 0.905 |
| UBC5 (AT1G63800.1) | G5309i6L1104 | RHC1A (AT2G40830.2) | G25218i1L563 | 0.901 |
| UBC5 (AT1G63800.1) | G5309i6L1104 | UBC7 (AT5G59300.1) | G70621i1L873 | 0.931 |
| UBC5 (AT1G63800.1) | G5309i6L1104 | UPL2 (AT1G70320.1) | G95075i1L308 | 0.912 |
| UBC7 (AT5G59300.1) | G70621i1L873 | RHC1A (AT2G40830.2) | G25218i1L563 | 0.912 |
| UBC7 (AT5G59300.1) | G70621i1L873 | UBC5 (AT1G63800.1) | G5309i6L1104 | 0.931 |
| UBC7 (AT5G59300.1) | G70621i1L873 | UPL2 (AT1G70320.1) | G95075i1L308 | 0.925 |
| UPL2 (AT1G70320.1) | G95075i1L308 | AT3G11630.1 | G18126i3L790 | 0.91 |
| UPL2 (AT1G70320.1) | G95075i1L308 | FER4 (AT2G40300.1) | G5051i5L1826 | 0.9 |
| UPL2 (AT1G70320.1) | G95075i1L308 | RHC1A (AT2G40830.2) | G25218i1L563 | 0.905 |
| UPL2 (AT1G70320.1) | G95075i1L308 | UBC5 (AT1G63800.1) | G5309i6L1104 | 0.912 |
| UPL2 (AT1G70320.1) | G95075i1L308 | UBC7 (AT5G59300.1) | G70621i1L873 | 0.925 |
