# Supplementary material for: Identification of key genes involved in secondary metabolite biosynthesis in Digitalis purpurea
Source: PLoS One. 2023 Mar 9;18(3):e0277293. doi: 10.1371/journal.pone.0277293 (PMC9997893; doi:10.1371/journal.pone.0277293)
Supplement: S11 Table — Annotation of each node in protein-protein interactions of hub proteins. (DOCX) [file pone.0277293.s013.docx]

**S11 Table. Annotation of hub protein interactions.** Annotation of each node in protein-protein interactions of hub proteins.

| **Accession Number** | **Annotation of node** |
| --- | --- |
| **AT2G19730.2**  (G22654i2L548) | Ribosomal L28e protein family; Its function is described as structural constituent of ribosome; Involved in translation, ribosome biogenesis; Located in cytosolic ribosome, ribosome, cytosolic large ribosomal subunit, chloroplast, plasma membrane; Expressed in 23 plant structures; Expressed during 14 growth stages; Contains the following InterPro domains: Ribosomal protein L28e (InterPro: IPR002672). |
| **AT3G10950.1**  (G13596i1L492/G73507i1L280) | Zinc-binding ribosomal protein family protein; Its function is described as structural constituent of ribosome; Involved in translation, ribosome biogenesis; Located in ribosome, cytosolic large ribosomal subunit; Contains the following InterPro domains: Ribosomal protein, zinc-binding domain (InterPro: IPR011332), Ribosomal protein L37ae (InterPro: IPR002674), Ribosomal protein L37ae/L37e, N-terminal domain (InterPro: IPR011331). |
| **AT3G11630.1**  (G18126i3L790) | 2-Cys peroxiredoxin BAS1, chloroplastic; Thiol-specific peroxidase that catalyzes the reduction of hydrogen peroxide and organic hydroperoxides to water and alcohols, respectively. Plays a role in cell protection against oxidative stress by detoxifying peroxides. May be an antioxidant enzyme particularly in the developing shoot and photosynthesizing leaf; Belongs to the peroxiredoxin family. AhpC/Prx1 subfamily. |
| **AT3G18740.1**  (G25740i1L487) | Ribosomal protein L7Ae/L30e/S12e/Gadd45 family protein; Its function is described as structural constituent of ribosome; Involved in translation; Located in cytosolic ribosome, cytosolic large ribosomal subunit; Expressed in 23 plant structures; Expressed during 13 growth stages; Contains the following InterPro domains: Ribosomal protein L7Ae/L30e/S12e/Gadd45 (InterPro: IPR004038), Ribosomal protein L30e (InterPro: IPR000231). |
| **AT4G28060.1**  (G19329i1L356) | Cytochrome c oxidase, subunit Vib family protein; This protein is one of the nuclear-coded polypeptide chains of cytochrome c oxidase, the terminal oxidase in mitochondrial electron transport. This protein may be one of the heme-binding subunits of the oxidase. |
| **COX2** (ATMG00160.1,  G105887i1L345) | Cytochrome c oxidase subunit 2; Cytochrome c oxidase is the component of the respiratory chain that catalyzes the reduction of oxygen to water. Subunits 1- 3 form the functional core of the enzyme complex. Subunit 2 transfers the electrons from cytochrome c via its binuclear copper A center to the bimetallic center of the catalytic subunit 1 (By similarity). |
| **FER4**  (AT2G40300.1,  G5051i5L1826) | Ferritin-4, chloroplastic; Encodes FERRITIN 4, AtFER4. Ferritins are a class of 24-mer multi-meric proteins found in all kingdoms of life. Function as the main iron store in mammals. Evidence suggests that Arabidopsis ferritins are essential to protect cells against oxidative damage, but they do not constitute the major iron pool. Localize to mitochondria. Knock out mutants are not sensitive to abiotic stress. |
| **RHC1A**  (AT2G40830.2,  G25218i1L563) | Probable E3 ubiquitin-protein ligase RHC1A; Probable E3 ubiquitin-protein ligase that may possess E3 ubiquitin ligase activity *in vitro*. |
| **UBC5**  (AT1G63800.1,  G5309i6L1104) | Ubiquitin-conjugating enzyme E2 5; Accepts the ubiquitin from the E1 complex and catalyzes its covalent attachment to other proteins. |
| **UBC7**  (AT5G59300.1,  G70621i1L873) | Ubiquitin-conjugating enzyme E2 7; Accepts the ubiquitin from the E1 complex and catalyzes its covalent attachment to other proteins. Involved in the formation of multiubiquitin chains. Signal the protein for selective degradation. |
| **UPL2**  (AT1G70320.1,  G95075i1L308) | Encodes a ubiquitin-protein ligase-like protein containing a HECT domain. There are six other HECT-domain UPLs in Arabidopsis. |
