# Supplementary material for: Identification of key genes involved in secondary metabolite biosynthesis in Digitalis purpurea
Source: PLoS One. 2023 Mar 9;18(3):e0277293. doi: 10.1371/journal.pone.0277293 (PMC9997893; doi:10.1371/journal.pone.0277293)
Supplement: Attachment — Submitted filename: Response to Reviewers.docx [file pone.0277293.s014.docx]

**Responses to comments of the Editor**

1. Please ensure that your manuscript meets PLOS ONE's style requirements, including those for file naming.

Answer

The manuscript was improved in accordance with the format of PLOS ONE. The main body, title page, and author affiliations are edited and corrected based on the format.

2. We suggest you thoroughly copyedit your manuscript for language usage, spelling, and grammar. If you do not know anyone who can help you do this, you may wish to consider employing a professional scientific editing service.

Answer

We thoroughly copyedited our manuscript for language usage, spelling, and grammar. In addition, an academic professional English editor edited our manuscript completely. Finally, we prepared a track-changed copy of our manuscript edited by a professional academic English editor as a separate file labeled 'New Manuscript' in supplementary information.

3. Upon resubmission, please provide the following: The name of the colleague or the details of the professional service that edited your manuscript

Answer

Our colleague Ms. Parisa Assayi (parisaassayi@email.com) who is an academic professional English editor paraphrased our manuscript and corrected it for language usage, spelling, and grammar.

4. A copy of your manuscript showing your changes by either highlighting them or using track changes (uploaded as a *supporting information* file). A clean copy of the edited manuscript (uploaded as the new *manuscript* file)

Answer

Finally, we prepared a track-changed copy of our manuscript edited by a professional academic English editor as a separate file labeled 'New Manuscript' in supplementary information.

5. We note that Figures 1 in your submission contain copyrighted images. All PLOS content is published under the Creative Commons Attribution License (CC BY 4.0), which means that the manuscript, images, and Supporting Information files will be freely available online, and any third party is permitted to access, download, copy, distribute, and use these materials in any way, even commercially, with proper attribution. For more information, see our copyright guidelines: http://journals.plos.org/plosone/s/licenses-and-copyright. We require you to either (1) present written permission from the copyright holder to publish these figures specifically under the CC BY 4.0 license, or (2) remove the figures from your submission:

Answer

We have removed the copyrighted section of Figure 1 from our submission and a new edited version was uploaded.

6. Please include captions for your Supporting Information files at the end of your manuscript, and update any in-text citations to match accordingly. Please see our Supporting Information guidelines for more information: <http://journals.plos.org/plosone/s/supporting-information>.

Answer

We have included captions for our Supporting Information files at the end of our manuscript in the part of Supporting Information.

7. Please review your reference list to ensure that it is complete and correct. If you have cited papers that have been retracted, please include the rationale for doing so in the manuscript text, or remove these references and replace them with relevant current references. Any changes to the reference list should be mentioned in the rebuttal letter that accompanies your revised manuscript. If you need to cite a retracted article, indicate the article’s retracted status in the References list and also include a citation and full reference for the retraction notice.

Answer

We have rechecked our reference list and have complied with the **Vancouver format**. We have not cited any retracted reference. In addition, we did not add or remove any reference as all of editions to the reference list have been mentioned in the revised manuscript.

**Responses to comments of the Reviewer #1**

1. In page 2, line 17, 140 hub genes mentioned. Whereas in the very first page it was mentioned 20 hub genes, please check

Answer

Actually, we have chosen seven modules among 34 modules and identified top 20 hub genes in each of module. Therefore, a total of 140 hub genes were identified from these seven selected modules.

2. Quality of some figures are really bad, please improve them, few figures are added in reverse orientation

Answer

The quality of all figures was improved and corrected based on the format with PACE. The font inside the figures has also been set to Arial of 8-12. In addition, the orientation of the figures was changed based on the comment.

3. I could not see Supplementary Table S1, the given RAR folder was corrupted.

Answer

The S1 Table was presented in a new corrected Supplementary Information file.

**Responses to comments of the Reviewer #2**

1- Why the authors have chosen so many tissue? Does secondary metabolites be expressed in all the tissues?

Answer

These tissues were required for performing WGCNA analysis. Therefore, more tissue types help us to find more reliable correlations for finding the genes effective on metabolite production. It makes it possible to obtain the common network genes involved in biosynthesis of secondary metabolites in different tissues. According to S1 Table, secondary metabolites be produced in all the tissues but in different amounts.

2- Authors must validate few candidate genes with real-time PCR analysis to confirm the RNA-seq data obtained from online resources.

Answer

Thank you for your precise comment. Actually, we have tried a lot to complete this experiment but unfortunately we have been encountered with the COVID-19 pandemic and then our lab was forbidden for all of communications. Now days, we got into the financial troubles.

3- Authors have just compiled the online based data. No study to confirm any dataset was done.

Answer

Thank you for your comment. As mentioned in the previous comment we not succeed to confirm our *in silico* analysis in lab using real-time PCR analysis cause of the restrictions. On the other hand, we have tried to cover this defeat using other online famous and comprehensive databases.

4- Tables does not need to put as main text. They can be supplied as supplementary data.

Answer

All tables have supplied as supplementary data.

5- What is novel in the study?

Answer

Cardiac glycosides are plant-made compounds mostly by members of the *Digitalis* genus, like *Digitalis* *purpurea*. *D.* *purpurea* (for digitoxin) is one of the major sources of the cardiac glycosides most frequently employed in medicine. In spite of the clinical significance of medicinal plants, hardly ever a comprehensive study on biosynthesis of metabolites has been considered. In this study, for the first time, an attempt has been made to identify key genes and pathways associated with biosynthesis of secondary metabolites based on a systemic view and combination of transcriptome and metabolome data.
