# Supplementary material for: Identification of key genes involved in secondary metabolite biosynthesis in Digitalis purpurea
Source: PLoS One. 2023 Mar 9;18(3):e0277293. doi: 10.1371/journal.pone.0277293 (PMC9997893; doi:10.1371/journal.pone.0277293)
Supplement: Attachment — Submitted filename: Response to Reviewers.docx [file pone.0277293.s015.docx]

**Responses to additional comments of the Editor**

Authors need to address the comments of the Reviewer 2. I think the validation of a few genes via RT-qPCR does not require substantial funds. Thus, authors should validate at least a few of the important genes highlighted in the study.

**Answer**

Thank you for your comments. Actually, we validated some key genes using RT-qPCR and addressed the comments of the Reviewer 2.

**Responses to the comments of the Reviewer #2**

Reviewer #2: The authors can take additional time to do the required essential experiments i.e., RT-PCR to confirm whether your plant behaves in the similar manner or act differentially.

**Answer**

Thank you for your comment. Based on this comment, after integrative data analysis, we validated some candidate key genes that are related to the biosynthesis of main secondary metabolites using RT-qPCR.
